# Ocular Involvement in Friedreich Ataxia Patients and Its Relationship with Neurological Disability, a Follow-Up Study

**DOI:** 10.3390/diagnostics10020075

**Published:** 2020-01-29

**Authors:** Pilar Rojas, Ana I. Ramírez, Rosa de Hoz, Manuel Cadena, Antonio Ferreras, Blanca Monsalve, Elena Salobrar-García, José L. Muñoz-Blanco, José L. Urcelay-Segura, Juan J. Salazar, José M. Ramírez

**Affiliations:** 1 Hospital General Universitario Gregorio Marañón, Instituto Oftálmico de Madrid, 28007 Madrid, Spain; pilar.rojas.lozano@gmail.com (P.R.); cadenamd@gmail.com (M.C.); blanca_monsalve@hotmail.com (B.M.); joseluis.urcelay@salud.madrid.org (J.L.U.-S.); 2 Instituto de Investigaciones Oftalmológicas Ramón Castroviejo, Universidad Complutense de Madrid, 28040 Madrid, Spain; airamirez@med.ucm.es (A.I.R.); rdehoz@med.ucm.es (R.d.H.); elenasalobrar@med.ucm.es (E.S.-G.); 3 Departamento de Inmunología, Oftalmología y ORL, Facultad de Óptica y Optometría, Universidad Complutense de Madrid, 28037 Madrid, Spain; 4 Hospital Universitario Miguel Servet, Instituto Aragonés de Ciencias de la Salud, 50009 Zaragoza, Spain; aferreras@msn.com; 5 Departamento de Inmunología, Oftalmología y ORL, Facultad de Medicina, Universidad Complutense de Madrid, 28040 Madrid, Spain; 6 Instituto de Investigación Sanitaria Gregorio Marañón, Unidad ALS-Neuromuscular, Departamento de Neurología, 28007 Madrid, Spain; joseluis.munoz@madrid.org

**Keywords:** OCT, Friedreich ataxia, FRDA, SARA, neurodegeneration, neurological disability, visual field

## Abstract

Background: This study compared functional and structural visual changes in Friedreich ataxia (FRDA) patients with healthy controls (HC) and correlated these changes with neurological disability. Methods: Eight FRDA Spanish patients and eight HC were selected from 2014 to 2018. Best corrected visual acuity (BCVA), visual field (VF), optic coherence tomography (OCT), and neurological disability measured by “scale for the assessment and rating of ataxia” (SARA) were taken in a basal exploration and repeated after 6 months. A linear mixed analysis and Bonferroni *p*-value correction were performed. Results: FRDA baseline and follow-up patients showed statistically significant decreases in BCVA, VF, and OCT parameters compared with the HC. Some of the VF measurements and most of the OCT parameters had an inverse mild-to-strong correlation with SARA. Moreover, the analysis of the ROC curve demonstrated that the peripapillary retinal nerve fiber layer (pRNFL) average thickness was the best parameter to discriminate between FRDA patients and HC. Conclusions: The follow-up study showed a progression in OCT parameters. Findings showed a sequential effect in pRNFL, ganglion cell complex (GCC), and macula. The VF and the OCT could be useful biomarkers in FRDA, both for their correlation with neurological disease as well as for their ability to evaluate disease progression.

## 1. Introduction

Friedreich ataxia (FRDA) is the most common autosomal recessive hereditary ataxia. It is a neurodegenerative disease produced by mutations in GAA triplet expansion in the first intron of the frataxin (*FXN*) gene on chromosome 9 (9q13-q1.1) [1,2,3,4,5,6]. The *FXN* gene encodes frataxin, a long protein targeted to the inner mitochondrial membrane, whose amount is greatly reduced by the triplet expansion [7]. This mutation results in an abnormal influx of iron into the mitochondria, which increases the susceptibility of the nervous system, including the visual pathway, to oxidative stress [8,9,10]. The incidence of FRDA is between 1 in 30,000–50,000 people and it is more common in Caucasians [4,5,8,11,12]. The disease onset is typically before the age of 25 and does not demonstrate a gender predilection [3,4].

Clinically, frataxin deficiency is characterized by spinocerebellar and sensory ataxia with the absence of deep tendon reflexes, dysarthria, hypertrophic cardiomyopathy, and scoliosis. Further possible additional features include diabetes mellitus, *pes cavus*, hypoacusia or deafness, optic atrophy, and eye movement abnormalities [5,8,13]. These ocular motor anomalies reflect the disruption of the brainstem–cerebellar circuit and include saccadic dysmetria, disrupted pursuit, and vestibular abnormalities, the most common manifestation of which is a fixation instability with frequent square-wave jerks [8,10,13,14,15,16,17,18,19]. In addition, other visual ophthalmic manifestations have been described in up to 30% of patients, including optic neuropathy, the involvement of the optic radiation, and, less commonly, a retinitis pigmentosa-like syndrome [8,20]. Visual field defects in FRDA patients range from severe visual field impairment to isolated regions of reduced sensitivity [2,8]. Thus, most patients with FRDA present a slowly progressive degenerative process involving both the optic nerve and optic radiations, as documented by the diffusion-weighted imaging (DWI) investigation [8]. However, in a few cases, all of those who had severe disease and large triplet expansion can suffer from a subacute/acute visual failure mimicking Leber hereditary optic neuropathy (LHON) [21,22].

Most mitochondrial diseases, such as LHON and dominant optic atrophy (DOA), have a preferential involvement of the small axons that form the papillo-macular bundle—the anatomical substrate for central and color vision and high spatial frequency contrast sensitivity [9,23,24]. Nevertheless, in FRDA, the papillo-macular bundle seems to be preserved [8,9,10,20]. Thus, visual acuity loss is uncommon [8].

Retinal ganglion cell (RGC) death is a specific target for mitochondrial-mediated neurodegeneration in the retina [9,22,23,24]. RGC and their axons can be analyzed by optic coherence tomography (OCT). OCT is a noninvasive imaging technique which provides major, more precise, and reproducible information, that years ago could only be obtained by funduscopy [8]. It is also sensitive to the decrease of the ganglion cell complex (GCC) and peripapillary retinal nerve fiber layer (pRNFL) thickness when visual impairment is not clinically apparent [8,9]. There are a few studies about FRDA and OCT [2,8,20,25]. In addition, these studies correlate RNFL and macular thickness with visual acuity, contrast sensitivity, neurological disability, and the duration of the disease. However, these studies are not longitudinal.

The aims of this study were (i) to study best corrected visual acuity (BCVA), the visual field (VF), macular thickness (MT), GCC, and pRNFL thickness (including quadrants and sectors) by OCT in FRDA patients; (ii) to compare these results with healthy controls (HC); this was a follow-up study and had two explorations: the baseline and the follow-up after 6 months; and (iii) the correlation of these visual changes with neurological disability measured by Scale for the Assessment and Rating of Ataxia (SARA) [26].

## 2. Material and Methods

### 2.1. Participants

This prospective longitudinal study was carried out at the Gregorio Marañón Hospital and Ramón Castroviejo Ophthalmologic Research Institute in the Complutense University of Madrid, Spain from 2014 to 2018. The study protocol, which adhered to the tenets of the Helsinki Declaration, was approved by the Gregorio Marañón Hospital Ethical Committee (NCT03285204, 15 September 2017) (available online: https://clinicaltrials.gov/ct2/show/NCT03285204, accessed on 29 January 2020). Each subject included in the study gave a written informed consent before entering the study.

FRDA-diagnosed and genetically confirmed patients were evaluated in both the Neurology and the Ophthalmology Department of Gregorio Marañón Hospital. All FRDA patients needed wheelchairs because all of them had spinocerebellar and sensory ataxia with the absence of deep tendon reflexes. Other problems they had were dysarthria, hypertrophic cardiomyopathy, scoliosis, hypoacusia, or fixation instability. Sixteen eyes from 8 FRDA Spanish patients were selected and compared with 16 eyes from 8 healthy controls. The Spanish HC group was selected from volunteers.

### 2.2. Study Protocol

All patients underwent an exhaustive complete neurological and general examination by a neurologist, including the “scale for the assessment and rating of ataxia” (SARA), which measures neurological disability in FRDA patients, ranging from 0 (minimum neurological disability) up to 40 points (maximum neurological disability) [26]. For the ophthalmological examination, both eyes of each patient were analyzed. All participants met the following inclusion criteria: (i) being free of ocular disease; (ii) Age-Related Eye Disease Study (AREDS) Clinical Lens Standards < 2; (iii) being free of systemic disorders affecting vision (except FRDA); (iv) having less than ±5 sphere-cylindrical refractive error; and (v) having intraocular pressure of less than 20 mmHg.

For screening, all FRDA patients and control subjects underwent a complete ophthalmologic examination, including assessment of BCVA, refraction, anterior segment biomicroscopy, Goldman applanation tonometer (AT900, Haag--Streit, Köniz, Switzerland), dilated fundus examination, and a spectral-domain Cirrus HD-OCT Model 4000 (Carl Zeiss Meditec, Dublin, CA, USA; software version 6.2), using an Optic Disc 200 × 200 and a Macular Cube and 512 × 128 scanning protocols [27,28]. MT, GCC, and pRNFL thickness were measured by OCT Cirrus after pupil dilation. The mean values were considered for statistical analysis. All tests were performed by the same ophthalmologist under the same conditions in a baseline evaluation (FRDA and control baseline groups, respectively), and at the follow-up after 6 months (FRDA and control follow-up groups, respectively).

As in the Early Treatment Diabetic Retinopathy Study (ETDRS) [27], MT data were displayed in three concentric rings centered in the fovea. These rings were distributed as follows: a central macular thickness (CMT) with a diameter of 1 mm; an inner macular ring (IMR) with a diameter of 3 mm, and an outer macular ring (OMR) with a diameter of 6 mm. In addition, the inner and outer rings were each divided into four quadrants (superior, inferior, nasal, and temporal). The total volume of the macula, as provided by the OCT, was also used. The GCC covers two inner layers of the retina: IPL (inner plexiform layer) and GCL (ganglion cell layer), which were measured by special segmentation software provided by Cirrus, checked by the ophthalmologist and re-centered if needed. The pRNFL average thickness was measured. pRNFL was also segmented into 4 quadrants (superior, temporal, inferior and nasal), and in 12 clock hours (H) (H3 as nasal, H6 as inferior, H9 as temporal, and H12 as superior in a right eye). Left eyes were converted to right eyes with a specular image [29]. The analyzed area was centered manually, and the absence of segmentation errors was confirmed for each scan. The desirable scan criteria were determined as the signal-to-noise ratio >7/10. All measurements were given in microns (µm), according to the calibration provided by the manufacturers and the total volume in mm^3^.

VFs were analyzed by a Humphrey Field Analyzer 750i (Humphrey Zeiss Systems, Dublin, CA, USA; 24-2) with SITA (Swedish Interactive Threshold Algorithm) Fast 24-2 strategy. Mean deviation (MD), pattern standard deviation (PSD), and visual field index (VFI) were used as perimetric indices of generalized sensitivity loss, localized scotomas, and the percentage of useful residual vision of the patient, respectively. False-positive (FP) and false-negative (FN) percentages were taken into account as reliability parameters.

### 2.3. Statistical Analysis

Data for the statistical analysis were introduced and processed in SPSS 23.0 (SPSS Inc.©, IBM Corporation, Somers, NY, USA) and StataCorp. 2015 (Stata Statistical Software: Release 14. College Station, TX: StataCorp LLC, College Station, TX, USA).

Minimum sample size was calculated in seven eyes for each group (ratio of sample sizes in the control/FRDA groups = 1) on the basis of a difference in the average pRNFL thickness of 25.1 microns and standard deviations (SD) of 10.3 and 10.9, a type 1 error rate of 0.05, and a power of 95% (MedCalc software). Difference of pRNFL thickness was based on the study of Ferreras et al. [30].

The normality of the distribution was assessed with the Kolmogorov–Smirnov test, and data did not follow a normal distribution. A mixed linear analysis was used, both in the baseline and in the follow-up analysis, in order not to overestimate the statistical power of the results because both eyes of the patients were considered for the study. For the follow-up variables, a mixed linear analysis was also applied, using the time as a variable too. We performed four comparisons as follows: control baseline vs. control follow-up, FRDA baseline vs. FRDA follow-up, control baseline vs. FRDA baseline, and control follow-up vs. FRDA follow-up.

To correlate functional and anatomical values with neurological disability measured by SARA, a Spearman rho test was employed. Moreover, an ROC curve was also performed.

Data are reported as mean values ± SD. All *p*-values were corrected using Bonferroni correction. A *p*-value of <0.05 was considered statistically significant.

## 3. Results

The demographic and clinical data of FRDA patients and the control group are shown in Table 1. The mean time from diagnosis in the FRDA group was 220.50 ± 147.55 months.

The comparison between control baseline vs. control follow-up did not show statistical differences in any visual parameters measured in our study.

### 3.1. Scale for the Assessment and Rating of Ataxia (SARA)

There was a significant difference in neurological disability measured by SARA between control baseline (0.00 ± 0.00) vs. FRDA baseline (28.38 ± 3.54); and control follow-up (0.00 ± 0.00) vs. FRDA follow-up (29.38 ± 4.32). A *p*-value < 0.001 was found in both cases (Table 1).

### 3.2. Best-Corrected Visual Acuity (BCVA)

Significant differences were found in the following comparisons: control baseline (0.99 ± 0.03) vs. FRDA baseline (0.68 ± 0.23); control follow-up (0.98 ± 0.04) vs. FRDA follow-up (0.63 ± 0.23), *p* < 0.001 was found in both cases; and FRDA baseline (0.68 ± 0.23) vs. FRDA follow-up (0.63 ± 0.23), *p* < 0.05 (Table 1).

### 3.3. Visual Field (VF)

In terms of the VF parameters, there were statistically significant differences both in control baseline vs. FRDA baseline and in control follow-up vs. FRDA follow-up. In both cases, VFI, MD, and PSD parameters were decreased in FRDA patients. In addition, FRDA patients had a higher rate of FN and FP with respect to HC, but visual fields were reliable (Table 1).

Three different common patterns of VF effect were found: the first pattern showed reduced sensitivity in a paracentral area, the second showed superior and/or inferior concentric arcuate defects, and the third pattern showed a general and concentric reduction of sensitivity.

The VFI and the MD had a moderate inverse correlation with SARA in the baseline exploration (VFI = −0.564 (*p* = 0.035) and MD = −0.554 (*p* = 0.040)) (Table 1).

### 3.4. Optical Coherence Tomography (OCT)

The comparison between control baseline vs. control follow-up did not show statistical differences in the OCT.

### 3.5. Peripapillary Retinal Nerve Fiber Layer (pRNFL)

**Figure 1:**
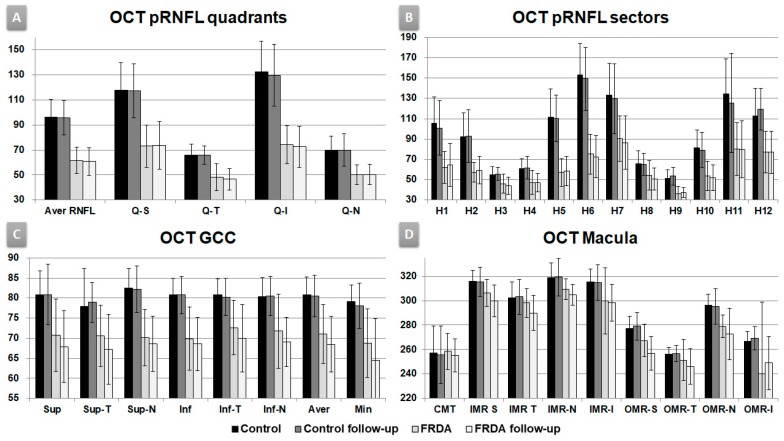
Optic coherence tomography outcomes, follow-up study. Vertical axis in microns. (**A**) OCT pRNFL (peripapillary retinal nerve fiber layer) quadrants. Aver: average, Q: quadrants, S: superior, T: temporal, I: inferior, N: nasal. (**B**) OCT pRNFL horary sectors. H: clock-hour position. (**C**) OCT macula. CMT: central macular thickness. IMR: inner macular ring; OMR: outer macular ring; S: superior, T: temporal, N: nasal, I: inferior. (**D**) OCT GCC (ganglion cell complex). Sup: superior, Inf: inferior, T: temporal, N: nasal, Aver: average, Min: minimum.

Control baseline vs. FRDA baseline and control follow-up vs. FRDA follow-up: there was a statistically significant decrease in the pRNFL average thickness, all quadrants and all horary sectors in FRDA patients (*p* < 0.05 in all instances, Table 2, Figure 1A,B), except in H8 in control baseline vs. FRDA baseline. In addition, H7 was the only sector significantly decreased in FRDA follow-up patients in comparison with FRDA baseline (*p* = 0.05).

Peripapillary RNFL parameters had a significant inverse mild–strong correlation with SARA in average pRNFL thickness (baseline = −0.562, follow-up = −0.658), pRNFL quadrants: temporal (baseline = −0.749, follow-up = −0.803) and inferior (baseline = −0.672, follow-up = −0.647); and pRNFL sectors: H7 (baseline = −0.808, follow-up = −0.681), H8 (baseline = −0.562, follow-up = −0.633), H9 (baseline = −0.684, follow-up = −0.585), H10 (baseline = −0.786, follow-up = −0.783), and H11 (baseline = −0.682, follow-up = −0.621) (*p* < 0.05 in all instances, Table 2).

**Figure 2:**
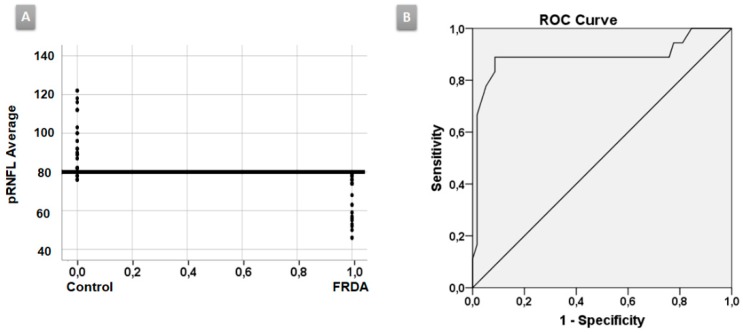
ROC curve. (**A**) The average peripapillary retinal nerve fiber layer (pRNFL) thickness was the best parameter to discriminate between Friedreich ataxia (FRDA) patients and healthy controls (HC) with an area under the curve (AUC) of 0.984. (**B**) The cut-off point was 80.5 microns.

ROC curve: In the baseline FRDA group, the average pRNFL thickness was the best parameter to discriminate between FRDA and HC with an area under the curve (AUC) = 0.984, the cut-off point of which was 80.5 microns (sensibility = 100%, specificity = 87.5%) (Figure 2A,B).

### 3.6. Ganglion Cell Complex (GCC)

Control baseline vs. FRDA baseline and control follow-up vs. FRDA follow-up: there was a statistically significant decrease in all GCC areas in FRDA patients (*p* ≤ 0.05 in all instances, Table 3, Figure 1C), except in supero-temporal area in control baseline vs. FRDA baseline.

FRDA baseline vs. FRDA follow-up: there was a statistically significant decrease in the FRDA follow-up group in superior and supero-temporal, infero-temporal GCC areas, and GCL average and minimum thickness (*p* < 0.05 in all instances, Table 3, Figure 1C).

All GCC parameters had a significant moderate inverse correlation with SARA in the baseline and follow-up explorations, except the infero-nasal area (*p* < 0.05 in all instances) (Table 3).

### 3.7. Macula

The comparison between control baseline vs. FRDA baseline showed a statistically significant decrease in FRDA patients in (i) superior and nasal in OMR areas; (ii) cube volume, and (iii) cube average thickness (*p* < 0.05 in all instances, Table 4, Figure 1D).

In addition, the comparison between control follow-up vs. FRDA follow-up displayed a statistically significant decrease in FRDA patients in (i) nasal and inferior IMR; (ii) superior, nasal, and inferior OMR areas; (iii) cube volume, and (iv) cube average thickness (*p* < 0.05 in all instances, Table 4, Figure 1D).

In FRDA baseline vs. FRDA follow-up comparison, there was a statistically significant decrease in the FRDA follow-up group in superior, temporal, and nasal IMR areas and superior OMR area (*p* < 0.05 in all instances, Table 4, Figure 1D).

Macula parameters had mild inverse correlations with SARA which were significant in (i) superior (baseline = −0.507, follow-up = −0.634) and temporal (baseline = −0.501, follow-up = −0.620) IMR; (ii) OMR superior (follow-up = −0.643); (iii) cube volume (follow-up = −0.518), and (iv) cube average thickness (follow-up = −0.518) (*p* < 0.05 in all instances, Table 4).

## 4. Discussion

In the present study, it was demonstrated that both functional (BCVA, VF) and structural (OCT) visual measures correlated with neurological disability in FRDA patients in both the baseline and follow-up study.

FRDA is a mitochondrial disease, but contrary to other mitochondrial diseases such as LHON [31], significant loss of BCVA appears at a late stage. This late loss of visual acuity has been observed in our baseline study as in others [2,8,20,25] and this decrease continues in our follow-up study.

Regarding VF in FRDA patients, as in previous studies [2,8], we also found three patterns of VF effect, which ranged from a reduced sensitivity in a paracentral area, followed by superior and/or inferior concentric arcuate defects, leading to a general and concentric reduction of sensitivity until the late stages. Thus, in most FRDA patients, the central vision was spared early in the disease, but eventually, it deteriorated with the progression of the disease. In addition, we have also analyzed the correlation between VF and neurological disability. In our patients, the visual function index and the mean deviation had a mild inverse correlation with SARA score. Therefore, visual function was well correlated with neurological disability in FRDA.

There are very scarce studies using OCT in FRDA patients [2,8,20,25]; however, our study is the only one that includes a follow-up. In our study, most of the pRNFL parameters were decreased in FRDA patients compared with HC, both in baseline and in follow-up explorations. According to other authors we observed, there was a statistically significant decrease in pRNFL average thickness in the baseline group compared with HC. The previous studies analyzed the average pRNFL, but only Seyer et al. [25] analyzed the pRNFL quadrants. Our findings were similar to theirs; thus, there was a statistically significant decrease in all pRNFL quadrants with respect to controls (from a greater to a lesser degree of affectation: inferior, superior, nasal, and temporal, the latter corresponds to the papillo-macular bundle). This is the only study in which the pRNFL was studied using horary sectors. In our study, all pRNFL horary sectors were statistically decreased with respect to HC both in baseline and in follow-up explorations, except H8 in the comparison control baseline vs. FRDA baseline. On the other hand, in comparison with FRDA baseline patients, a significant decrease has been demonstrated in sector H7 in follow-up FRDA, which could correspond to a progression of the disease. The study of the pRNFL by sectors could be important because there is a certain degree of organization of retinal ganglion cell axons in the nerve fiber layer (retinotopic distribution) [32,33,34]. Moreover, OCT pRNFL thickness parameters (average pRNFL thickness, temporal and inferior quadrants, and sector H7–H11) had a mild–strong inverse correlation with neurological disability in baseline and follow-up explorations (Table 2). The temporal quadrant and sector H8—which correspond to the papillo-macular bundle (parvocellular system)—had effect to a lesser degree, which could explain why BCVA was not affected until late stages in FRDA patients. The predominant involvement of the superior and inferior pRNFL quadrants indicate a preferential contribution of parasol retinal ganglion cells projecting to the magnocellular pathway (M-cells), which are mainly located in the extramacular retina and do not specifically contribute to BCVA [35]. Finally, the analysis of the ROC curve demonstrated that the pRNFL average thickness was the best parameter to discriminate between FRDA patients and controls with a cut-off point of 80.5 µ (AUC = 0.984). Thus, values less than this number would correspond to FRDA patients (Figure 2A,B).

In our study, the control baseline vs. FRDA baseline comparison showed a normal foveal thickness and a significant decrease in macular thickness (OMR superior and nasal, cube volume, and cube average thickness (Table 4; Figure 1D)). However, Noval et al. [20] reported a normal foveal thickness and macular volume. This difference with respect to our study could be related to the disease onset. The population of Noval was much younger and with fewer years of evolution (ranged 7–204 months with an average of 21.1 ± 39.89 months [20]) than ours (ranged 72–369 with an average of 220.50 ± 147.55 months). On the contrary, Dag et al. [2] reported a reduction in foveal thickness, and Seyer et al. [25] found a reduction in macular thickness values. Both authors correlated the decrease of these parameters with disease duration. In our study, we also found a correlation between macular thickness and neurological disability by not only analyzing foveal thickness and macular volume, but also the macula by areas. We found an inverse significant mild correlation in IMR superior and temporal, both in baseline and in follow-up explorations; and OMR superior, cube volume, and cube average thickness in the follow-up exploration (Table 3). Therefore, the higher the SARA score (the greater the neurological disability), the lower the macular thickness.

To the best of our knowledge, there are no follow-up studies in FRDA patients. Among our patients in the follow-up cohort, we found that the decrease in macular thickness values progressed significantly in different sectors (superior, temporal, and nasal in IMR and superior in OMR areas). This decrease means that, over time, the disease affects the macula. With respect to GCC, Dag et al. [2] were the only authors who analyzed the GCC and reported that the mean GCC thickness in the superior and inferior macula were significantly lower in FRDA patients. In our study, we divided the superior and inferior regions of the macula into six areas. All the GCC areas (except supero-temporal) displayed a statistically significant decrease in FRDA patients in the baseline exploration. In the follow-up study, GCC thickness was significantly decreased in all areas in the FRDA follow-up cohort with respect to HC. In addition, in most GCC areas there was a significant decrease in FRDA follow-up patients with respect to baseline, which could correspond to a progression of the disease (Table 3). This decrease could be due to a general effect of retinal ganglion cells, which are specific targets for mitochondrial-mediated neurodegeneration [8,22,23,24]. In this disease, the first effect of the ganglion cells could be exerted on the axons, where there are more mitochondria.

Therefore, a decrease in the pRNFL thickness is the primary event observed in these patients. Secondly, the ganglion cell body loss could be due to the death of injured axons. This circumstance could produce a late decrease in macular thickness. Thus, FRDA may affect ganglion cell axons as well as ganglion cell somas because of retinal axonopathy as well as neuronopathy [25]. These data suggest that macular alterations in the OCT (1) may be a feature of the most advanced FRDA, (2) could be responsible for the visual loss in these patients [25], and (3) could help evaluate FRDA progression.

In conclusion, although FRDA is a mitochondrial disease, its behavior in the retina and optic nerve is not typical of these diseases (such as LOHN or DOA). In FRDA patients, both in baseline and in follow-up, the macular, GCC, and pRNFL thicknesses, as determined by OCT, were decreased compared with healthy controls, presumably because of damage to the ganglion cell axons and posteriorly, the loss of retinal ganglion cells. In addition, VFI, MD, and many OCT parameters are strongly inversely correlated with neurological disability, especially in pRNFL. Moreover, the analysis of the ROC curve demonstrated that the pRNFL average thickness was the best parameter to discriminate between FRDA patients and controls. The follow-up study has allowed us to describe the sequential effects in FRDA: first in the pRNFL, then in the GCC, and finally, in the macula. Visual field, and especially OCT, could be good biomarkers in FRDA patients, because of both their correlation with neurological disease as well as their ability to evaluate disease progression.

## Figures and Tables

**Table 1:** Demographic data, SARA, and visual field.

Gender (Male/Female)	Control		FRDA		*p*-Value				Spearman Correlation SARA	*p*-Value Spearman Correlation	Spearman Correlation SARA Follow-Up	*p*-Value Spearman Correlation Follow-Up
	Baseline	Follow-Up	Baseline	Follow-Up	Control Baseline vs. Control Follow-Up *	Control Baseline vs. FRDA Baseline *	Control Follow-up vs. FRDA Follow-Up *	FRDA Baseline vs. FRDA Follow-Up *				
	*n* = 16	*n* = 16	*n* = 16	*n* = 16								
	(4/4)	(4/4)	(4/4)	(4/4)								
	Mean ± SD											
**BCVA**	0.99 ± 0.03	0.98 ± 0.04	0.68 ± 0.23	0.63 ± 0.23	0.317	**<0.001**	**<0.001**	**0.040**	(−0.292)	0.273	(−0.424)	0.102
**Age**	45.50 ± 11.28	45.50 ± 11.67	36.13 ± 10.46	37.00 ± 10.85	1.000	0.091	0.188	0.080	0.108	0.691	0.000	1.000
**IOP**	16.00 ± 1.89	15.31 ± 1.89	14.13 ± 2.75	14.00 ± 2.56	0.095	0.058	0.079	0.766	0.095	0.708	0.005	0.808
**SARA**	0.00 ± 0.00	0.00 ± 0.00	28.38 ± 3.54	29.38 ± 4.32	1.000	**<0.001**	**<0.001**	0.071	------	------	------	------
**VFI**	98.88 ± 1.59	99.13 ± 1.09	66.57 ± 34.61	70.50 ± 25.80	0.425	**0.039**	**0.026**	0.860	(−0.564)	**0.035**	(−0.284)	0.371
**MD**	(−0.40) ± 1.67	(−0.05) ± 1.09	(−13.88) ± 10.67	(−10.22) ± 9.21	0.353	**0.005**	**0.006**	0.233	(−0.554)	**0.040**	(−0.272)	0.392
**PSD**	1.61 ± 0.45	1.68 ± 0.62	4.13 ± 2.51	4.48 ± 3.14	0.494	**0.005**	**0.034**	0.308	0.188	0.520	0.201	0.532
**% FN**	0.94 ± 1.34	2.19 ± 3.04	14.86 ± 10.60	12.83 ± 14.08	0.054	**0.002**	0.107	0.959	0.270	0.351	0.029	0.929
**% FP**	3.49 ± 3.20	3.25 ± 4.07	6.36 ± 7.47	13.75 ± 14.91	0.846	0.299	0.123	**0.035**	0.173	0.554	0.253	0.428

* Lineal mixed model. Data are expressed as mean ± standard deviation (SD) (except gender). Numbers in bold *p* < 0.05 after Bonferroni correction. BCVA: best-corrected visual acuity; IOP: intraocular pressure. FRDA: Friedreich ataxia; SARA: Scale for the Assessment and Rating of Ataxia; parameters that measure retinal sensitivity: VFI: Visual Field Index; MD: mean deviation (dB); PSD: pattern standard deviation; parameters that measure visual field reliability: % FN: percentage of false-negative errors; % FP: percentage of false-positive errors.

**Table 2:** Analysis of peripapillary RNFL measures by OCT between FRDA and control group.

Peripapillary Analysis (µm)		Control		FRDA		*p*-Value				Spearman Correlation SARA	*p*-Value Spearman Correlation	Spearman Correlation SARA Follow-Up	*p*-Value Spearman Correlation Follow-Up
		Baseline	Follow-Up	Baseline	Follow-Up	Control Baseline vs. Control Follow-Up *	Control Baseline vs. FRDA Baseline *	Control Follow-Up vs. FRDA Follow-Up *	FRDA Baseline vs. FRDA Follow-Up *				
		*n* = 16	*n* = 16	*n* = 16	*n* = 16								
		Mean ± SD											
**Average RNFL thickness**		96.38 ± 14.25	95.69 ± 13.82	61.56 ± 10.51	60.75 ± 11.20	0.556	**<0.001**	**<0.001**	0.359	(−0.562)	**0.024**	(−0.658)	**0.006**
**pRNFL quadrants (µm)**	**Superior**	117.56 ± 22.22	117.38 ± 21.54	73.06 ± 17.04	73.75 ± 18.90	0.888	**<0.001**	**<0.001**	0.708	(−0.425)	0.100	(−0.444)	0.085
	**Temporal**	65.94 ± 8.79	65.88 ± 7.21	48.25 ± 10.89	46.75 ± 8.60	0.964	**<0.001**	**<0.001**	0.251	(−0.749)	**<0.001**	(−0.803)	**<0.001**
	**Inferior**	132.50 ± 24.46	129.75 ± 24.70	74.31 ± 15.27	72.5 ± 16.54	0.076	**<0.001**	**<0.001**	0.406	(−0.672)	**0.004**	(−0.647)	**0.007**
	**Nasal**	69.69 ± 11.44	69.94 ± 12.92	50.25 ± 7.84	50.25 ± 8.06	0.890	**<0.001**	**<0.001**	1.000	0.051	0.852	(−0.358)	0.173
**pRNFL sectors (µm)**	**H1**	105.25 ± 26.43	100.94 ± 26.74	62.19 ± 15.79	64.5 ± 20.93	0.080	**0.001**	**0.007**	0.382	(−0.219)	0.415	(−0.097)	0.720
	**H2**	92.38 ± 23.31	92.63 ± 25.95	57.19 ± 9.66	59.25 ± 13.52	0.942	**<0.001**	**<0.001**	0.322	(−0.063)	0.818	(−0.329)	0.213
	**H3**	54.63 ± 7.93	55.75 ± 6.59	46.00 ± 9.20	44.25 ± 8.40	0.349	**0.002**	**<0.001**	0.422	0.112	0.680	(−0.351)	0.183
	**H4**	60.81 ± 9.45	61.75 ± 10.99	47.19 ± 11.88	46.81 ± 9.05	0.378	**<0.001**	**0.001**	0.881	0.028	0.917	(−0.302)	0.256
	**H5**	111.44 ± 27.70	110.38 ± 22.80	57.06 ± 13.59	58.5 ± 14.63	0.686	**<0.001**	**<0.001**	0.622	(−0.290)	0.277	0.016	0.952
	**H6**	153.38 ± 30.21	149.19 ± 30.94	75.19 ± 19.41	72.63 ± 20.77	0.071	**<0.001**	**<0.001**	0.503	(−0.370)	0.158	(−0.229)	0.393
	**H7**	133.50 ± 31.04	129.50 ± 34.27	90.56 ± 22.12	86.38 ± 20.06	0.234	**0.003**	**0.003**	**0.050**	(−0.808)	**<0.001**	(−0.681)	**0.004**
	**H8**	65.69 ± 12.66	65.13 ± 11.04	54.25 ± 14.28	50.75 ± 10.87	0.798	0.089	**0.010**	0.120	(−0.562)	**0.024**	(−0.633)	**0.009**
	**H9**	51.38 ± 8.08	53.38 ± 8.80	36.43 ± 6.77	37.44 ± 4.56	0.463	**0.001**	**<0.001**	0.461	(−0.684)	**0.004**	(−0.585)	**0.017**
	**H10**	81.19 ± 17.84	79.19 ± 17.05	53.63 ± 14.66	51.94 ± 12.8	0.195	**<0.001**	**<0.001**	0.285	(−0.786)	**<0.001**	(−0.783)	**<0.001**
	**H11**	134.38 ± 34.44	125.50 ± 48.73	80.31 ± 25.96	79.69 ± 28.01	0.230	**<0.001**	**0.005**	0.826	(−0.682)	**0.004**	(−0.621)	**0.010**
	**H12**	113.00 ± 26.62	119.13 ± 20.47	76.94 ± 20.51	76.94 ± 21.02	0.273	**<0.001**	**<0.001**	1.000	(−0.030)	0.913	(−0.241)	0.368

* Lineal mixed model. Data are expressed as mean ± standard deviation (SD). Numbers in bold *p* < 0.05 after Bonferroni correction. FRDA: Friedreich ataxia. H: clock-hour position; pRNFL: retinal nerve fiber layer; C/D: cup-to-disc; SARA: Scale for the Assessment and Rating of Ataxia).

**Table 3:** Analysis of ganglion cell complex measures by OCT between FRDA and control group.

GCC Analysis (µm)		Control		FRDA		*p*-Value				Spearman Correlation SARA	*p*-Value Spearman Correlation
		Baseline	Follow-Up	Baseline	Follow-Up	Control Baseline vs. Control Follow-Up *	Control Baseline vs. FRDA Baseline *	Control Follow-Up vs. FRDA Follow-Up *	FRDA Baseline vs. FRDA Follow-Up *		
		*n* = 16	*n* = 16	*n* = 16	*n* = 16						
		Mean ± SD									
	**Central**	80.75 ± 5.97	80.88 ± 7.62	70.75 ± 8.99	67.88 ± 8.94	0.922	**0.006**	**0.002**	**0.003**	(−0.616)	**0.011**
**Superior**	**Temporal**	77.94 ± 9.43	78.94 ± 4.92	70.56 ± 7.61	67.25 ± 8.70	0.528	0.104	**0.001**	**<0.001**	(−0.568)	**0.022**
	**Nasal**	82.50 ± 4.90	82.13 ± 5.81	70.19 ± 7.01	68.63 ± 6.87	0.459	**<0.001**	**<0.001**	0.132	(−0.568)	**0.017**
**Inferior**	**Central**	80.75 ± 4.22	80.75 ± 4.67	69.88 ± 7.82	68.56 ± 6.62	1.000	**<0.001**	**<0.001**	0.379	(−0.537)	**0.032**
	**Temporal**	80.81 ± 3.99	80.25 ± 4.68	72.63 ± 6.81	69.94 ± 8.39	0.361	**<0.001**	**0.001**	**<0.001**	(−0.679)	**0.004**
	**Nasal**	80.38 ± 4.69	80.50 ± 4.90	71.75 ± 9.22	69.06 ± 6.07	0.848	**0.002**	**<0.001**	0.189	(−0.460)	0.073
**GCL (µm) thickness**	**Average**	80.81 ± 4.43	80.56 ± 5.20	71.06 ± 7.33	68.5 ± 6.90	0.611	**0.001**	**<0.001**	**<0.001**	(−0.547)	**0.028**
	**Minimum**	79.06 ± 4.22	78.06 ± 5.70	68.75 ± 8.55	64.5 ± 10.37	0.182	**0.004**	**0.002**	**0.004**	(−0.519)	**0.040**

* Lineal mixed model. Data are expressed as mean ± standard deviation (SD). Numbers in bold *p* < 0.05 after Bonferroni correction. FRDA: Friedreich ataxia; OCT: optical coherence tomography; GCC: ganglion cell complex; GCL: ganglion cell layer; SARA: Scale for the Assessment and Rating of Ataxia; µm: microns.

**Table 4:** Analysis of macular thickness measures by OCT between FRDA and control group.

Macular Analysis (µm)		Control		FRDA		*p*-Value				Spearman Correlation SARA	*p*-Value Spearman Correlation	Spearman Correlation SARA Follow-Up	*p*-Value Spearman Correlation Follow-Up
		Baseline	Follow-Up	Baseline	Follow-Up	Control Baseline vs. Control Follow-Up *	Control Baseline vs. FRDA Baseline *	Control Follow-Up vs. FRDA Follow-Up *	FRDA Baseline vs. FRDA Follow-Up *				
		*n* = 16	*n* = 16	*n* = 16	*n* = 16								
		Mean ± SD											
**Fovea o CMT**		257.00 ± 22.32	255.81 ± 23.61	258.63 ± 14.79	255.25 ± 13.53	0.364	0.250	0.453	0.129	(−0.158)	0.558	0.038	0.890
**IMR**	**Superior**	316.06 ± 8.60	315.31 ± 12.06	306.25 ± 11.07	299.94 ± 12.97	0.645	0.188	0.056	**<0.001**	(−0.507)	**0.045**	(−0.634)	**0.008**
	**Temporal**	302.38 ± 12.95	303.06 ± 14.21	298.06 ± 11.93	289.88 ± 14.36	0.594	0.945	0.265	**<0.001**	(−0.501)	**0.016**	(−0.620)	**0.010**
	**Nasal**	318.69 ± 12.34	319.13 ± 15.15	309.31 ± 8.34	304.63 ± 8.52	0.709	0.256	**0.050**	**0.001**	(−0.179)	0.507	(−0.029)	0.916
	**Inferior**	315.50 ± 10.17	314.88 ± 14.58	299.75 ± 26.87	298.25 ± 14.88	0.787	0.191	**0.043**	0.832	(−0.462)	0.072	(−0.316)	0.234
**OMR**	**Superior**	277.31 ± 9.95	279.06 ± 11.38	267.44 ± 13.20	256.88 ± 13.75	0.350	**0.027**	**<0.001**	**0.001**	(−0.373)	0.155	(−0.643)	**0.007**
	**Temporal**	256.00 ± 5.54	256.63 ± 6.42	251.19 ± 16.81	246.06 ± 14.62	0.720	0.557	0.101	0.069	(−0.007)	0.978	(−0.359)	0.172
	**Nasal**	296.19 ± 8.91	295.38 ± 14.39	278.88 ± 9.29	272.69 ± 21.13	0.771	**<0.001**	**0.003**	0.165	(−0.278)	0.297	(−0.351)	0.183
	**Inferior**	266.81 ± 8.10	269.31 ± 9.44	240.38 ± 58.36	248.93 ± 21.73	0.137	0.092	**0.001**	0.548	(−0.452)	0.079	(−0.335)	0.204
**Cube Volume**		9.96 ± 0.35	10.04 ± 0.29	9.34 ± 0.66	9.07 ± 0.99	0.246	**0.023**	**0.001**	0.254	(−0.258)	0.335	(−0.518)	**0.040**
**Cube average thickness**		276.50 ± 9.55	279.06 ± 7.83	259.81 ± 18.64	251.94 ± 27.5	0.157	**0.026**	**0.001**	0.237	(−0.285)	0.285	(−0.518)	**0.040**

* Lineal mixed model. Data are expressed as mean ± standard deviation (SD). Numbers in bold *p* < 0.05 after Bonferroni correction. FRDA: Friedreich ataxia; OCT: optical coherence tomography; CMT: central macular thickness; IMR: inner macular ring; OMR: outer macular ring; SARA: Scale for the Assessment and Rating of Ataxia.
